# Generalizing findings from a randomized controlled trial to a real-world study of the *iLookOut*, an online education program to improve early childhood care and education providers’ knowledge and attitudes about reporting child maltreatment

**DOI:** 10.1371/journal.pone.0227398

**Published:** 2020-01-08

**Authors:** Chengwu Yang, Carlo Panlilio, Nicole Verdiglione, Erik B. Lehman, Robert M. Hamm, Richard Fiene, Sarah Dore, David E. Bard, Breanna Grable, Benjamin Levi

**Affiliations:** 1 Department of Epidemiology and Health Promotion, New York University College of Dentistry, New York City, New York, United States of America; 2 Department of Educational Psychology, Counseling and Special Education, Pennsylvania State University, University Park, Hershey, Pennsylvania, United States of America; 3 Departments of Humanities, Penn State College of Medicine, Hershey, Pennsylvania, United States of America; 4 Departments of Population Health Sciences, Penn State College of Medicine, Hershey, Pennsylvania, United States of America; 5 Department of Family and Preventive Medicine, University of Oklahoma Health Sciences Center, Oklahoma City, Oklahoma, United States of America; 6 Departments of Psychology & Human Development Research Center, Pennsylvania State University, Middletown, Pennsylvania, United States of America; 7 Research Institute for Key Indicators, Middletown, Pennsylvania, United States of America; 8 Department of Pediatrics, University of Oklahoma Health Sciences Center, Oklahoma City, Oklahoma, United States of America; 9 Departments of Humanities & Pediatrics, Penn State College of Medicine, Hershey, Pennsylvania, United States of America; East Carolina University Brody School of Medicine, UNITED STATES

## Abstract

In recent years, real-world studies (RWS) are gaining increasing interests, because they can generate more realistic and generalizable results than randomized controlled clinical trials (RCT). In 2017, we published a RCT in 741 early childhood care and education providers (CCPs). It is the Phase I of our *iLookOut for Child Abuse* project (*iLookOut*), an online, interactive learning module about reporting suspected child maltreatment. That study demonstrated that in a RCT setting, the *iLookOut* is efficient at improving CCPs’ knowledge of and attitudes towards child maltreatment reporting. However, the generalizability of that RCT’s results in a RWS setting remains unknown. To address this question, we design and conduct this large RWS in 11,065 CCPs, which is the Phase II of the *iLookOut*. We hypothesize replication of the earlier RCT findings, i.e., the *iLookOut* can improve CCPs’ knowledge of and attitudes toward child maltreatment reporting in a real world setting. In addition, this RWS also explores whether demographic factors affect CCPs’ performance. Results of this RWS confirmed the generalizability of the previous RCT’s results in a real world setting. It yielded similar effect sizes for knowledge and attitudes as were found in the earlier RCT. Cohen’s d for knowledge improvement was 0.95 in that RCT, 0.96 in this RWS; Cohen’s d for attitude improvement was 0.98 in that RCT, 0.80 in this RWS. Also, we found several significant differences in knowledge and attitude improvement with regard to age, race, education, and employment status. In conclusion, *iLookOut* improves knowledge and attitudes of CCPs about child maltreatment prevention and reporting in a real-world setting. The generalizability of the initial RCT findings to this RWS provides strong evidence that the *iLookout* will be effective in other real world settings. It can be a useful model for other interventions aimed at preventing child maltreatment.

Clinical trial registration for the original RCT: NCT02225301 (ClinicalTrials.gov Identifier)

## Introduction

While randomized controlled trials (RCT) have long been seen as the “gold standard” for evaluating the efficacy of interventions, there are well-known limitations to their generalizability [1]. Accordingly, there have been growing interests in real-world studies (RWS) to generate real-world evidence (RWE) that are more realistic and generalizable [2–9], and RWE is increasingly valued by regulators and payers [10]. In addition, RWE and the RCT can happily co-exist and complement each other [9].

Recently, we published data from an RCT about the online educational intervention, the *iLookOut for Child Abuse* (*iLookOut*), showing that it improved early childhood care and education providers (CCPs) knowledge and attitudes about child maltreatment and its reporting [11]. In this follow-up study, through an RWS, we evaluate whether these results are generalizable to a broad population of CCPs in a real-world setting.

There are more than 675, 000 confirmed cases of child maltreatment annually in the United States [12], but less than 1% of these are reported by CCPs (U.S. DHHS, 2017). This extremely low report rate by CCPs is alarming, given the fact that about 12 million U.S. children are served in some form of a child care setting, that children five years-old or younger account for 46% of confirmed maltreatment and more than 75% of maltreatment-related deaths (U.S. DHHS, 2017), and that the true incidence of child maltreatment is likely much higher than currently detected [13, 14]. Such underreporting suggests a need for CCPs to become better prepared to protect young children from maltreatment by improving their knowledge and attitude towards child maltreatment reporting. As has been identified by the Institute of Medicine and others, a key obstacle to improving awareness and reporting is the lack of evidence-based interventions [15–17]. In addition, the US Preventive Services Task Force (USPSTF) recently called for more evidence-based primary care interventions to prevent child maltreatment [12]. Several small studies have evaluated in-person training for CCPs [18, 19], and a brief online intervention [20, 21]. However, large studies involving scalable interventions are still lacking.

To meet this need, we created *iLookout*, an interactive online learning program designed specifically for CCPs (https://ilookoutproject.org/). An initial RCT using a test and re-test design with 741 participants demonstrated the feasibility of this three-hour online training, as well as its efficacy at increasing knowledge and changing attitudes about child maltreatment and its reporting [11]. Though this initial trial was promising, with large Cohen’s d effect sizes for knowledge (0.95) and attitudes (0.98), its generalizability was limited by several factors, notably the potential for selection bias. Participants were enrolled only if the director of the child care program responded to the recruitment mailing. Family- and home-based CCP programs were under-represented, as were racial and ethnic minorities. In addition, enrollment was limited to a four-week period in early summer. Also, the sample size limited the opportunity for in-depth comparisons among subgroups.

To address these limitations, the present RWS used a statewide, open-enrollment design to enlist a larger, more representative sample of CCPs. We hypothesized that *iLookOut*’s efficacy at increasing knowledge and attitudes would be confirmed in this real-world sample, and our exploratory aim was to evaluate the impact of key demographic characteristics.

## Materials and methods

### Design

The Penn State College of Medicine Institutional Review Board approved this study prior to its initiation (IRB #: 1243). This RWS employed an open enrollment, single group, pre- and post-test design. Participants completed a demographic questionnaire, as well as previously validated knowledge and attitude measures regarding child maltreatment and its reporting [11]. Given the observational feature of this RWS, we have ensured that the manuscript adheres to the appropriate Equator Network guidelines, such as the STROBE (*Strengthening the Reporting of Observational Studies in Epidemiology)* Statement [22].

### Participants

As an open-enrollment RWS, participants were not actively recruited to this study. However, all mandated reporters in Pennsylvania (including CCPs) are required by law to complete a mandated reporter training, and *iLookOut* was one of more than a dozen state-approved trainings listed on Pennsylvania’s Department of Human Services website, and was available online at no charge. As such, online searches and word of mouth were the means for dissemination. Participant data reported here are from CCPs who completed *iLookOut* between January 2015 and March 2018. CCPs provided online informed consent prior to participating, and earned three hours of professional development credit for completing the learning program. No other incentives or remuneration were provided.

### Intervention

The *iLookOut* online learning program uses an interactive, video-based storyline in which the learners take the role of a teacher of 4–5 year-olds at a child care facility. As key events unfold through interactions involving children, parents, and co-workers (all played by actors), the learners have to decide how to best respond. At different points, learners are posed questions. Based on their answer, they are provided didactic material to educate them about various aspects of child maltreatment. Other times, the learners must choose how to respond to events in the story. Throughout the learning program, CCPs can access multiple resource files covering definitions of maltreatment, facts about maltreatment, red flags, etc.[11].

### Measures

The pre- and post-test comprise two parts. The first is a 21-item, true or false, expert-validated instrument previously described [11]. It measures individuals’ knowledge about what constitutes child maltreatment, risk factors for maltreatment, and legal requirements for reporting suspected maltreatment. Correct answer to each of the 21 true or false items is scored as 1 point, and wrong answer is scored as 0 point. Therefore, the total score of the knowledge scale ranges from 0 to 21, which higher score representing more knowledge about child maltreatment. The second part contains 13 items, rated on 7-point Likert-style scales, from a previously validated instrument [23] adapted to comport with Pennsylvania jurisdictional standards. It measures individuals’ attitude towards reporting potential child maltreatment. An individual’s attitude score is the average score of the 13 items, ranges from 1 to 7, with higher score representing more positive attitude towards reporting potential child maltreatment. The pre- and post-test question items were identical, but to minimize recall bias, their sequencing orders were changed between the pre- and the post-test.

### Sample size and statistical analysis

Given the RWS nature of this study, no a priori sample size estimation was planned. However, post-hoc power analyses were implemented to check the statistical power for some important subgroup analyses [4]. We also compared participant demographics between the initial RCT and this RWS.

As with the RCT, the statistical analysis of this RWS examined *iLookOut*’s impact on CCPs’ knowledge and attitudes related to child maltreatment and its reporting. The two primary outcome variables were the total knowledge score and the total attitude score, both measured as “change”, i.e., total score at post-test minus at pre-test. The analysis focused on whether the present RWS confirmed the results of the initial RCT. To compare effect sizes between the RCT and the RWS, we used two measures: 1) the absolute difference, i.e., the measured change in pre- to post-test score for the RWS, minus the measured change in initial RCT; and 2) the Cohen’s d calculation [24]. In addition, we explored the impact of demographic factors on these two primary outcome variables through analysis of covariance (ANCOVA), framing demographic variables as covariates, and adjusting for pre-measurement scores. These demographic variables include age, gender, race/ethnicity, education, employment, parent/guardian status, prior trained status, work environment, years as practitioner, primary job responsibilities, and religiosity. We used the SAS software package, version 9.4, for statistical analyses, and the G*Power software package, version 3.1.9, for post-hoc power analyses.

## Results

During the 38 months of the RWS reported here, 11,605 CCPs completed the *iLookOut* online training. Compared to those CCPs in the initial RCT, these RWS participants were more representative of the general population of CCPs in Pennsylvania, particularly for its enrollment of Blacks (20.8% vs. 8.0%) and males (10.9% vs. 2.3%). In addition, the CCPs in this RWS were younger (48.0% vs. 40.4% aged below 30), and a greater proportion worked in more urban area (36.4% vs. 22.1%). Table 1 illustrates comparisons of full demographics between these two studies.

**Table 1:** Comparisons of demographic characteristics of early childcare professionals.

		Phase II: RWS	Phase I: RCT	Difference	p-value
Sample Size		11,065	741	10,324	
Age	18–29	5309 (48.0%)	299 (40.4%)	7.6%	<0.001
	30–44	2912 (26.3%)	216 (29.1%)	-2.8%	
	45+	2844 (25.7%)	226 (30.5%)	-4.8%	
Gender	Male	1210 (10.9%)	17 (2.3%)	8.6%	<0.001
	Female	9855 (89.1%)	724 (97.7%)	-8.6%	
Race/Ethnicity	Non-Hispanic White	7605 (68.7%)	624 (84.2%)	-15.5%	<0.001
	Non-Hispanic Black	2296 (20.8%)	59 (8.0%)	12.8%	
	Hispanic	658 (6.0%)	25 (3.4%)	2.6%	
	Asian	227 (2.1%)	15 (2.0%)	0.1%	
	Other	279 (2.4%)	18 (2.4%)	0.0%	
Education	Below High School	82 (0.7%)	0 (0.0%)	0.7%	<0.001
	High School or GED	4611 (41.7%)	197 (26.6%)	15.1%	
	Child Development Associate (CDA)	765 (6.9%)	101 (13.6%)	-6.7%	
	Associates	1483 (13.4%)	149 (20.1%)	-6.7%	
	Bachelors	2983 (27.0%)	229 (30.9%)	-3.9%	
	Masters or Doctoral	1141 (10.3%)	65 (8.8%)	1.5%	
Employment	Permanent Full-Time	6276 (56.7%)	534 (72.1%)	-15.4%	<0.001
	Permanent Part-Time	2943 (26.6%)	169 (22.8%)	3.8%	
	Contract for special services	177 (100.0%)	0 (0.0%)	100.0%	
	Substitute Teacher	206 (1.9%)	6 (0.8%)	1.1%	
	Seasonal	793 (7.2%)	28 (3.8%)	3.4%	
	Volunteer	334 (3.0%)	0 (0.0%)	3.0%	
	Other	336 (4.6%)	4 (0.5%)	4.1%	
Parent/Guardian	Yes	6089 (55.0%)	452 (61.0%)	-6.0%	0.002
	No	4976 (45.0%)	289 (39.0%)	6.0%	
Prior Trained	Yes	7371 (66.6%)	582 (78.5%)	-11.9%	<0.001
	No	3694 (33.4%)	159 (21.5%)	11.9%	
Work Environment	Rural	2191 (19.8%)	206 (27.8%)	-8.0%	
	Suburban	4848 (43.8%)	371 (50.1%)	-6.3%	<0.001
	Urban	4026 (36.4%)	164 (22.1%)	14.3%	
Years as Practitioner	<1	3272 (29.9%)	68 (9.2%)	20.7%	
	1–2	1652 (14.9%)	112 (15.1%)	-0.2%	<0.001
	3–5	2034 (18.4%)	145 (19.6%)	-1.2%	
	6–10	1657 (15.0%)	154 (20.8%)	-5.8%	
	11–15	887 (8.0%)	75 (10.1%)	-2.1%	
	>15	1563 (14.1%)	187 (25.2%)	-11.1%	
Primary job responsibilities	Teacher/caregiving staff (infant–grade 4)	7049 (63.7%)	555 (75.0%)	-11.3%	
	Early intervention specialist	184 (1.7%)	0 (0.0%)	1.7%	<0.001
	Support staff	651 (5.9%)	25 (3.4%)	2.5%	
	Director/Assistant Director	781 (7.1%)	95 (12.8%)	-5.7%	
	Other	2400 (21.7%)	66 (8.8%)	12.9%	
Religiosity	Extremely unreligious	198 (1.8%)	10 (1.4%)	0.4%	
	Unreligious	689 (6.2%)	54 (7.4%)	-1.2%	<0.001
	Somewhat unreligious	436 (3.9%)	13 (1.8%)	2.1%	
	Neutral	2568 (23.2%)	117 (15.9%)	7.3%	
	Somewhat religious	2503 (22.6%)	215 (28.8%)	-6.2%	
	Religious	4017 (36.3%)	287 (38.9%)	-2.6%	
	Extremely religious	654 (5.9%)	45 (5.9%)	0.0%	

Table 2 illustrates comparisons of the *iLookOut* training’s effect sizes on knowledge and attitude scores between this RWS and the RCT, demonstrating improved knowledge and attitudes about child maltreatment reporting for both studies. Pre- to post- changes in knowledge score increased by 2.80 for RWS participants, compared to 2.65 in the initial RCT, a 5.7% relative change. The Cohen’s d on the total knowledge score was 0.96 in this RWS versus 0.95 in the RCT, a 1% relative change. The pre-to post- change in attitude average score was 0.5 for RWS participants, versus 0.59 in the initial RCT, a -15.3% relative change. The Cohen’s d on the average attitude score was 0.80 in this RWS, versus 0.98 in the RCT, a relative change of -18.4%.

**Table 2:** Comparisons of effect sizes on knowledge and attitude scores.

	Post–Pre in Phase II: RWS	Post–Pre in Phase I: RCT	Difference: Phase II–Phase I	Relative Change	Cohen’s d in Phase II: RWS	Cohen’s d Phase I: RCT	Difference: Phase II–Phase 1	Relative Change
Knowledge: Total Score (Range: 0–21)	2.80 ± 2.90	2.65 ± 2.78	0.15	5.7%	0.96	0.95	0.01	1.0%
Attitude: Average Score (Range: 1–7)	0.50 ± 0.63	0.59 ± 0.60	-0.09	-15.3%	0.80	0.98	-0.18	-18.4%

Table 3 summarizes the results of exploratory multivariate analyses (ANCOVA) for each of the two outcome variables (knowledge and attitude scores) with all of the demographic variables. After adjustment for pre-measurement scores and all the other demographic variables, only four demographics (age, race, education, and employment) showed impacts on either of the two outcome variables, with age and education being positively correlated with increase in knowledge scores.

**Table 3:** Summary of analysis of covariance (ANCOVA) results.

	Total Knowledge Score (0–21)			Average Attitude Score (1–7)		
Variable**	Pre (Mean ± SD)	Post (Mean ± SD)	Mean Change (95% CI)*	Pre (Mean ± SD)	Post (Mean ± SD)	Mean Change (95% CI)*
Age						
18–29	13.7 ± 2.7	16.6 ± 2.9	2.9 (2.8, 2.9)	5.9 ± 0.7	6.4 ± 0.7	0.5 (0.5, 0.5)
30–44	14.1 ± 3.0	17.2 ± 2.9	3.2 (3.1, 3.3)	5.9 ± 0.8	6.4 ± 0.7	0.5 (0.5, 0.5)
More than 44	13.9 ± 3.1	17.4 ± 2.8	3.5 (3.4, 3.6)	5.8 ± 0.8	6.4 ± 0.7	0.5 (0.5, 0.5)
Gender						
Male	13.8 ± 3.0	16.9 ± 3.0	3.0 (2.8, 3.1)	5.9 ± 0.7	6.4 ± 0.7	0.5 (0.4, 0.5)
Female	13.9 ± 2.9	16.9 ± 2.9	3.1 (3.1, 3.2)	5.9 ± 0.7	6.4 ± 0.7	0.5 (0.5, 0.5)
Race						
White	14.0 ± 2.9	17.4 ± 2.8	3.5 (3.4, 3.5)	5.9 ± 0.7	6.4 ± 0.6	0.6 (0.5, 0.6)
Black or African American	13.5 ± 2.9	15.8 ± 2.9	2.2 (2.1, 2.3)	5.8 ± 0.8	6.2 ± 0.8	0.3 (0.3, 0.4)
American Indian or Alaska Native	13.5 ± 3.2	16.1 ±2.6	2.7 (1.9, 3.5)	6.1 ± 0.8	6.3 ± 0.7	0.4 (0.2, 0.5)
Hispanic	13.3 ± 2.8	16.0 ± 2.8	2.5 (2.4, 2.7)	5.9 ± 0.8	6.3 ± 0.7	0.4 (0.4, 0.5)
Asian	13.2 ± 3.4	16.0 ± 3.3	2.0 (1.7, 2.4)	5.8 ± 0.8	6.3 ± 0.7	0.4 (0.4, 0.5)
Native Hawaiian/ Pacific Islander	13.6 ± 3.6	16.1 ± 3.1	2.3 (1.1, 3.5)	5.6 ± 1.0	6.1 ± 1.1	0.4 (0.2, 0.7)
Other	13.2 ± 3.4	16.4 ± 3.3	2.9 (2.5, 3.2)	5.8 ± 0.8	6.3 ± 0.8	0.5 (0.4, 0.6)
Education						
8th Grade	12.8 ± 3.4	15.2 ± 3.2	1.7 (1.1, 2.2)	5.6 ± 0.9	6.0 ± 0.9	0.2 (0.1, 0.4)
High School Diploma or G.E.D.	13.4 ± 2.9	16.3 ± 3.0	2.6 (2.6, 2.7)	5.8 ± 0.8	6.3 ± 0.7	0.4 (0.4, 0.5)
Child Development Associate	14.0 ± 3.1	16.4 ± 2.9	2.6 (2.4, 2.8)	5.8 ± 0.8	6.3 ± 0.7	0.5 (0.4, 0.5)
Associate’s Degree	13.9 ± 2.9	16.8 ± 2.7	2.9 (2.8, 3.1)	5.9 ± 0.7	6.4 ± 0.7	0.5 (0.5, 0.5)
Bachelor’s Degree	14.2 ± 2.9	17.8 ± 2.7	3.8 (3.7, 3.9)	5.9 ± 0.7	6.5 ± 0.6	0.6 (0.6, 0.6)
Masters or Doctoral Degree	14.5 ± 3.0	18.0 ± 2.7	3.9 (3.7, 4.0)	6.0 ± 0.7	6.5 ± 0.6	0.6 (0.6, 0.6)
Employment						
Permanent full-time	14.0 ± 2.9	16.9 ± 2.9	3.0 (2.9, 3.0)	5.9 ± 0.7	6.4 ± 0.7	0.5 (0.5, 0.5)
Permanent part-time	13.5 ± 2.9	16.7 ± 3.0	3.1 (3.0, 3.2)	5.9 ± 0.8	6.3 ± 0.7	0.5 (0.5, 0.5)
Contract for special services/care	14.8 ± 2.4	18.0 ± 2.5	3.3 (2.9, 3.7)	6.0 ± 0.7	6.5 ± 0.6	0.5 (0.4, 0.6)
Substitute teacher	13.6 ± 3.1	17.3 ± 2.9	3.5 (3.1, 3.9)	6.0 ± 0.7	6.5 ± 0.7	0.5 (0.5, 0.6)
Seasonal or short-term	13.6 ± 2.7	17.4 ± 2.8	3.9 (3.7, 4.1)	5.9 ± 0.7	6.5 ± 0.6	0.6 (0.6, 0.7)
Volunteer	13.7 ± 3.2	17.7 ± 2.8	3.7 (3.4, 4.0)	5.9 ± 0.7	6.5 ± 0.6	0.6 (0.5, 0.6)
Other	13.5 ± 3.1	17.2 ± 2.9	3.4 (3.2, 3.7)	5.9 ± 0.7	6.4 ± 0.7	0.5 (0.4, 0.6)
Parent or guardian of child						
Yes	14.0 ± 3.0	17.0 ± 2.9	3.0 (3.0, 3.1)	5.9 ± 0.8	6.4 ± 0.7	0.5 (0.5, 0.5)
No	13.7 ± 2.8	16.9 ± 3.0	3.2 (3.1, 3.3)	5.9 ± 0.7	6.4 ± 0.7	0.5 (0.5, 0.5)
Previously trained						
Yes	14.2 ± 2.8	17.1 ± 2.8	3.1 (3.0, 3.1)	5.9 ± 0.7	6.4 ± 0.7	0.5 (0.5, 0.5)
No	13.1 ± 2.9	16.6 ± 3.0	3.2 (3.1, 3.3)	5.8 ± 0.8	6.3 ± 0.7	0.5 (0.5, 0.5)
Work Environment						
Rural	14.1 ± 2.9	17.1 ± 2.8	3.2 (3.1, 3.3)	5.9 ± 0.7	6.4 ± 0.7	0.5 (0.5, 0.5)
Suburban	13.8 ± 3.0	17.2 ± 2.9	3.3 (3.2, 3.4)	5.9 ± 0.7	6.4 ± 0.7	0.5 (0.5, 0.5)
Urban	13.7 ± 2.9	16.5 ± 3.0	2.9 (2.8, 3.0)	5.9 ± 0.8	6.3 ± 0.7	0.5 (0.5, 0.5)
Years as practitioner						
Less than 1	13.6 ± 2.8	16.9 ± 2.9	3.2 (3.1, 3.3)	5.9 ± 0.7	6.4 ± 0.7	0.5 (0.5, 0.5)
1–2	13.6 ± 2.9	16.7 ± 3.0	3.1 (3.0, 3.3)	5.9 ± 0.7	6.3 ± 0.7	0.5 (0.5, 0.5)
3–5	13.9 ± 2.9	16.8 ± 2.9	3.0 (2.9, 3.1)	5.9 ± 0.7	6.4 ± 0.7	0.5 (0.5, 0.5)
6–10	14.0 ± 3.0	17.1 ± 2.9	3.2 (3.0, 3.3)	5.8 ± 0.8	6.4 ± 0.7	0.5 (0.5, 0.5)
11–15	13.9 ± 3.2	17.0 ± 3.0	3.0 (2.8, 3.2)	5.8 ± 0.8	6.4 ± 0.7	0.5 (0.5, 0.5)
More than 15	14.3 ± 3.0	17.5 ± 2.8	3.0 (2.9, 3.2)	5.9 ± 0.8	6.4 ± 0.7	0.5 (0.5, 0.5)
Primary Job Responsibility						
Teacher/caregiving staff (age 0–5)	13.7 ± 2.9	16.8 ± 2.9	3.1 (3.1, 3.2)	5.9 ± 0.7	6.4 ± 0.7	0.5 (0.5, 0.5)
Early intervention specialist	14.7 ± 2.8	17.6 ± 2.9	2.9 (2.5, 3.3)	6.0 ± 0.7	6.4 ± 0.7	0.4 (0.3, 0.5)
Kindergarten teacher	13.1 ±3.0	16.6 ± 3.6	2.9 (2.5, 3.4)	5.7 ± 0.7	6.2 ± 0.8	0.4 (0.3, 0.5)
Early elementary teacher	13.5 ± 2.8	16.8 ± 3.1	3.1 (2.8, 3.3)	5.9 ± 0.7	6.4 ± 0.7	0.5 (0.5, 0.6)
Support staff	13.6 ± 2.8	16.6 ±2.9	3.0 (2.8, 3.2)	5.8 ± 0.7	6.4 ± 0.7	0.5 (0.5, 0.6)
Assistant Director	14.5 ± 2.9	17.2 ± 2.9	3.0 (2.7, 3.3)	5.9 ± 0.8	6.4 ± 0.6	0.5 (0.4, 0.6)
Director	15.0 ± 2.8	17.8 ± 2.6	3.1 (2.7, 3.3)	6.0 ± 0.7	6.5 ± 0.6	0.5 (0.4, 0.5)
Other	14.0 ± 2.9	17.3 ± 2.9	3.1 (3.0, 3.2)	5.9 ± 0.7	6.4 ± 0.7	0.5 (0.5, 0.5)
Religiosity						
Extremely Unreligious	14.4 ± 2.9	17.4 ± 3.0	3.3 (2.9, 3.6)	5.9 ± 0.7	6.4 ± 0.7	0.5 (0.4, 0.5)
Unreligious	13.9 ± 3.1	17.0 ± 2.9	3.1 (2.9, 3.3)	5.9 ± 0.7	6.4 ± 0.7	0.5 (0.5, 0.6)
Somewhat unreligious	14.1 ± 2.9	16.9 ± 2.8	3.0 (2.8, 3.3)	5.9 ± 0.7	6.4 ± 0.7	0.5 (0.5, 0.5)
Neutral	13.7 ± 2.9	16.5 ± 3.0	2.9 (2.8, 3.0)	5.9 ± 0.8	6.3 ± 0.7	0.5 (0.5, 0.5)
Somewhat religious	13.9 ± 2.9	17.0 ± 2.9	3.1 (3.0, 3.2)	5.9 ± 0.7	6.4 ± 0.7	0.5 (0.5, 0.5)
Religious	13.8 ± 3.0	17.0 ± 2.9	3.2 (3.1, 3.2)	5.9 ± 0.7	6.4 ± 0.7	0.5 (0.4, 0.5)
Extremely religious	13.9 ± 2.9	17.8 ± 2.8	3.7 (3.5, 3.9)	5.9 ± 0.7	6.5 ± 0.7	0.5 (0.5, 0.6)

* The mean changes come from a multivariable model for the change in the outcome adjusted for the pre-measurement and including all of the following demographic variables as covariates: age, gender, race/ethnicity, education, employment, parent/guardian status, prior trained status, work environment, years as practitioner, primary job responsibilities, and religiosity. As a result, the mean changes displayed are adjusted for all of the other variables.

** All of the variables have p-values less than 0.05, except for gender (p = 0.061), and primary job responsibilities (p = 0.641).

Post hoc power analysis indicates that with a total sample size of 11,065, at an alpha level of 0.05, with 80% power, the ANCOVA with 11 covariates would be able to detect an effect size as small as 0.04 among six groups; and using an effect size cut-off of 0.25, then the power would approach to 99.5%.

## Discussion

The results from this RWS demonstrate that in a large, representative sample of child care professionals (CCPs), the online *iLookOut* learning program is effective at improving knowledge and changing attitudes about child maltreatment and its reporting. These findings confirm the conclusions from the initial RCT of *iLookOut*, and demonstrate the feasibility of scaling this evidence-based, online mandated reporter training. This is notable insofar as more than 11,000 CCPs completed *iLookOut*, even when no special incentives were offered, and they reported being highly satisfied with the learning experience (paper forthcoming). No significant differences were identified with regard to CCPs’ parenting status, previous training, work environment, years as practitioner, primary job responsibility, or religiosity. However, age, race, education, and employment affected changes in knowledge or attitude scores, with older and more educated CCPs achieving increased gains in knowledge scores.

The generalizability of the initial RCT findings provides supporting evidence that the *iLookout* online learning program will be effective in other real world settings, and may be a useful model for other interventions aimed at preventing child maltreatment [12]. *iLookOut*’s general storyline and overall format are generalizable for all kinds of CCPs in all U.S. states, in part because state-specific information is housed in discrete learning modules (within the learning program) that can be readily adapted to comport with the laws and policies of different states. The efficacy of *iLookOut* does not appear to be affected by previous training, work environment, years as practitioner, primary job responsibility, parenting status, or religiosity. However, larger gains in knowledge were seen in CCPs who were older, more highly educated, employed seasonally, or white. More research is warranted to better understand the underpinnings of these differences, and how best to optimize gains in knowledge for all CCPs.

The statistical analyses reported here focus on effect sizes, instead of p-values, for several reasons. First, p-values are not a good measure of evidence [25]. Second, the misuse and maltreatment of p-values has led both researchers and the American Statistical Association to raise concerns about the limitations of p-value-driven conclusions [26, 27]. Third, the very large sample size (over 11,000) of this RWS could yield findings of statistical significance for even very small effect sizes that have no clinical significance [28]. Fourth, the large difference in sample size between the initial RCT and this RWS renders effect sizes a more meaningful comparison than p-values. Finally, for proposed sub-group analyses involving many demographic covariates, p-values are less likely to yield meaningful findings [29]. Accordingly, we compared effect sizes by examining the overlap of their confidence limits.

The present findings are limited by potential biases encountered in all RWS, including selection bias, information bias, and confounding [3]. Multivariate analysis (ANCOVA) was used to try to account for these factors, and the initial RCT does provide additional reassurance that the present findings are valid. However, without qualitative data, an explanatory model for the present findings will remain incomplete.

## Conclusion

This real-world study of more than 11,000 early childhood professionals (CCPs) who were neither recruited nor incentivized to complete the *iLookOut for Child Maltreatment* confirms that *iLookOut* significantly improves knowledge and attitudes regarding child maltreatment and its reporting. These results provide strong evidence that interactive, online interventions for helping prevent child maltreatment are both effective and scalable. A 5-year randomized controlled trial (https://clinicaltrials.gov/ct2/show/NCT02225301?term=NCT02225301&rank=1) is currently underway to evaluate how well *iLookOut* helps CCPs identify and report true child maltreatment.

## Supporting information

S1 Dataset (CSV)
